# Solitary atrial Rhabdomyoma in an infant with tuberous sclerosis: a case report and review of the literature

**DOI:** 10.1186/s12872-023-03639-4

**Published:** 2023-12-07

**Authors:** Ali Jawad, Zein Alabdin Hannouneh, Hadi Salame, Rida Jaber, Nader Eid

**Affiliations:** 1 Faculty of Medicine, Damascus University, Damascus, Syrian Arab Republic; 2 Faculty of Medicine, Al Andalus University for Medical Sciences, Tartus, Syrian Arab Republic; 3 Neonatology Intensive Care department, Damascus University Children Hospital, Damascus, Syrian Arab Republic

**Keywords:** Rhabdomyoma, Cardiac tumor, Tuberous sclerosis, Case report

## Abstract

**Background:**

Despite its rare incidence of 1/40,000, fetal cardiac rhabdomyoma (CR) represents the prevailing type of benign cardiac fetal tumors, which commonly affects the ventricles. Fetal CRs rarely occur in the right atrium. Thus, the presentation of atrial fibrillation and premature atrial contractions (PAC) due to a solitary cardiac rhabdomyoma is an extremely rare scenario. Our literature review found that only 2% (1 out of 61) of rhabdomyoma cases were found in the right atrium. The majority of fetal cardiac rhabdomyomas are associated with tuberous sclerosis complex (TSC).

**Case presentation:**

A 7-day-old male neonate presented with arrhythmias and an atrial mass for further evaluation. Echocardiography revealed a hyperechoic, round, uniform right atrial mass (25 mm). An abdominal and testicular ultrasound showed multiple thin-walled cortical cysts in both kidneys and a scrotal hydrocele, respectively. His laboratory workup was insignificant except for hypomagnesemia. Electrocardiography revealed junctional rhythm and PACs with wave distortions. A brain magnetic resonance imaging scan revealed multiple subependymal lesions on the frontal and occipital horns of the lateral ventricles. These findings (Fig. 1), along with a family history of TSC, confirmed the diagnosis of TSC with associated CR. The patient was treated symptomatically with an anti-convulsant and monitored with regular follow-ups. Surgical resection was not required.

**Conclusion:**

Despite CR’s predominance in the ventricles, a diagnosis of rhabdomyoma should be kept in mind in the presence of a solitary atrial mass and PACs. Physicians should evaluate systemic findings related to TSC and provide appropriate follow-up and family screening. Surgical resection is not always required, and symptom management can be achieved through medical treatment alone.

**Supplementary Information:**

The online version contains supplementary material available at 10.1186/s12872-023-03639-4.

## Background

Tuberous sclerosis complex (TSC) is an autosomal dominant condition that is distinguished by the presence of hamartomas in multiple organs throughout the body, including the heart [1]. Approximately 60% of pediatric patients diagnosed with TSC have cardiac rhabdomyomas (CR), whereas this cardiac manifestation is observed in only about 20% of adult individuals with TSC [2]. While fetal CRs are uncommon, they represent the prevailing type of cardiac fetal tumors. The incidence rate of CRs varies from 0.02 to 0.08% in live-born infants [3]. CRs are most commonly located in the right ventricle, followed by the interventricular septum, left ventricle, left atrium, and right atrium (RA) [4]. Echocardiography and cardiac magnetic resonance imaging (MRI) are essential imaging techniques employed in the diagnostic process [1]. Although most cases of fetal cardiac tumors are benign, the prognosis of the tumor depends on its location, size, and associated complications [5]. The treatment involves surgical resection in symptomatic cases with significant hemodynamic instability, whereas a thorough follow-up is required in asymptomatic cases [3] since CRs have a tendency to regress after the first year from birth [1].

Two previous literature reviews of all published cases with CRs and TSC-associated CRs were conducted up until 1990 and from 1990 to 2011, respectively [6, 7]. We conducted a PUBMED search from March 2011 to January 2023 and included all relevant case reports, case-series, and observational studies including CRs (Supplementary 1). We found a total of 88 cases, 65 (82%) of which were associated with TSC. The majority (73.8%) of rhabdomyomas occurred in the left ventricle (LV), 59% in the right ventricle, and only 2% (1 out of 61) in the RA. Around 58% (50 out of 86) had cardiac symptoms (arrythmias, bradycardia, tachycardia, cyanosis, hemodynamic instability, and murmurs), whereas 38% (22 out of 57) had neurologic symptoms (seizures, intellectual disability). Only 12% of cases required surgical intervention, whereas 79% underwent monitoring and sometimes needed medical therapy (everolimus, antiarrhythmics, and antipsychotics). On the other hand, 9% of cases (8 out of 85) died due to complications.

**Fig. 1:**
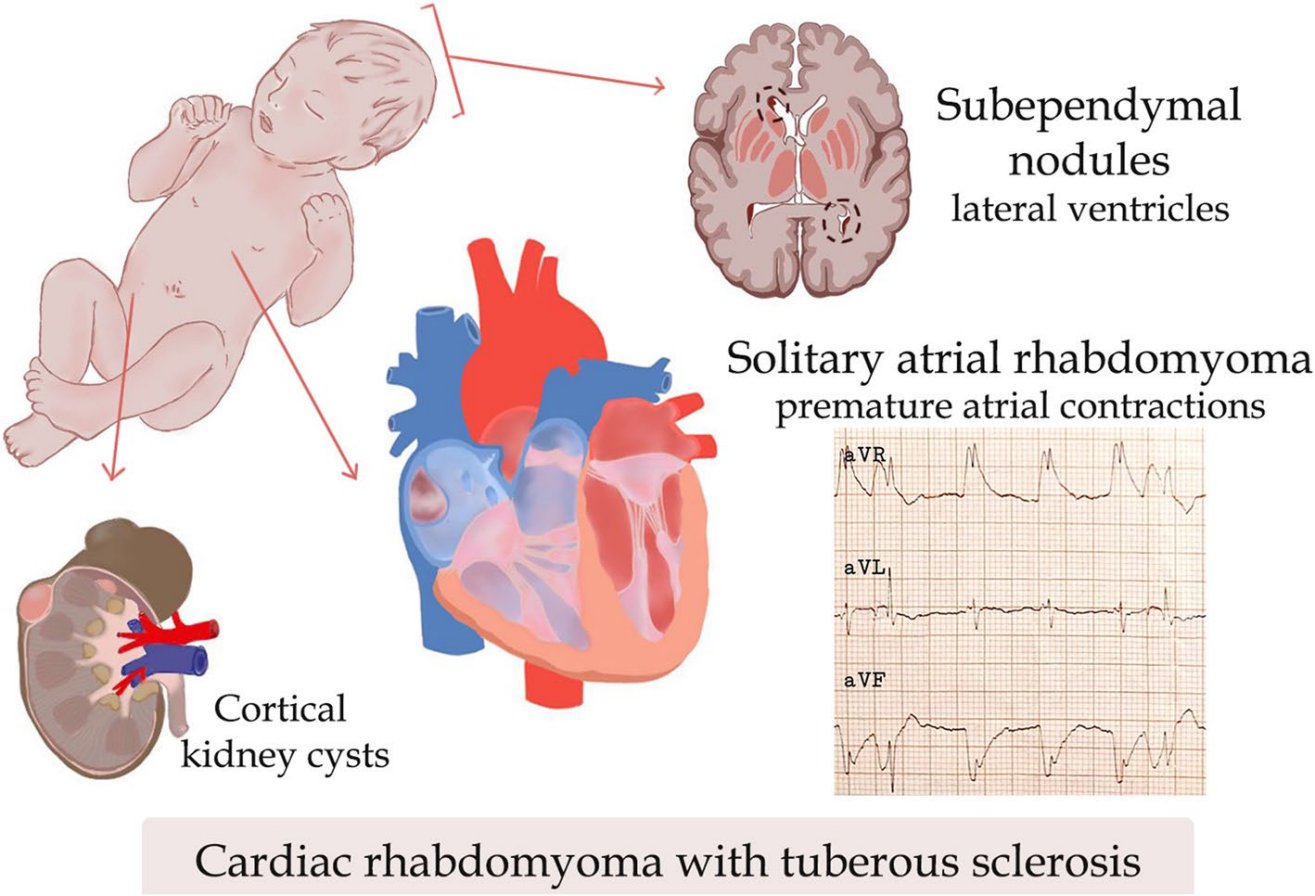
The presentation of a neonate with tuberous sclerosis

In this report, we present a rare presentation of PACs in a neonate due to a right atrial rhabdomyoma associated with TSC.

## Case presentation

A 7-day-old male neonate, born to non-consanguineous parents, gravida 3 para 2, at 39 weeks of gestation, cried immediately after birth and showed no signs of respiratory distress syndrome. He was referred to our department after an external clinic detected a solitary mass in the RA along with atrial fibrillation. He was initially asymptomatic and was admitted to the nursery for further care and monitoring of his cardiac condition. His family history included an older sibling who had been diagnosed with TSC at 2 years of age.

Upon admission, the chest and abdominal physical examinations were normal. Peripheral pulses were symmetrical, and heart rate was normal (120 bpm). An irregular rhythm was detected during auscultation.

**Fig. 2:**
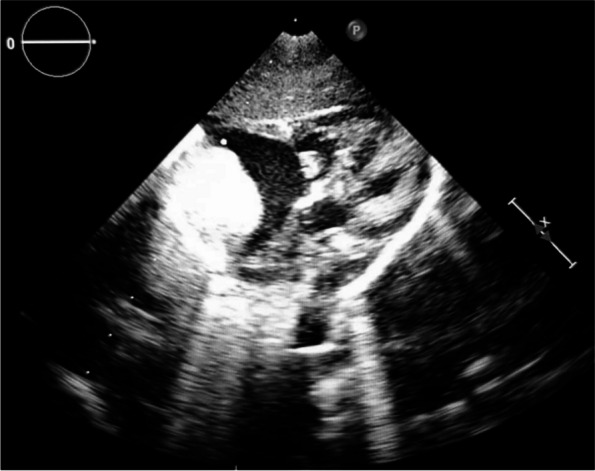
Echocardiogram revealing a hyperechoic, uniform, round 25 mm mass arising from the superolateral wall of the right atrium and protuding into the right atrial cavity, towards the tricuspid valve

Echocardiography showed a mildly dilated RA with a hyperechoic, homogenous mass measuring 25 × 20 mm in the superolateral wall of the RA with an outpouching that pointed towards the tricuspid valve (Fig. 2) (Supplementary 2). No other abnormalities were noted, and there were no signs of compression. Suspicion of CR was proposed. An additional abdominal and testicular ultrasound revealed multiple simple-appearing, thin-walled cysts located in the cortical layer of both kidneys. The largest cyst measured 6 × 4.3 mm. A scrotal hydrocele was also detected. Other findings were unremarkable. Additional evaluation by ophthalmoscopy identified bilateral temporal pallor of the optic nerve.

**Fig. 3:**
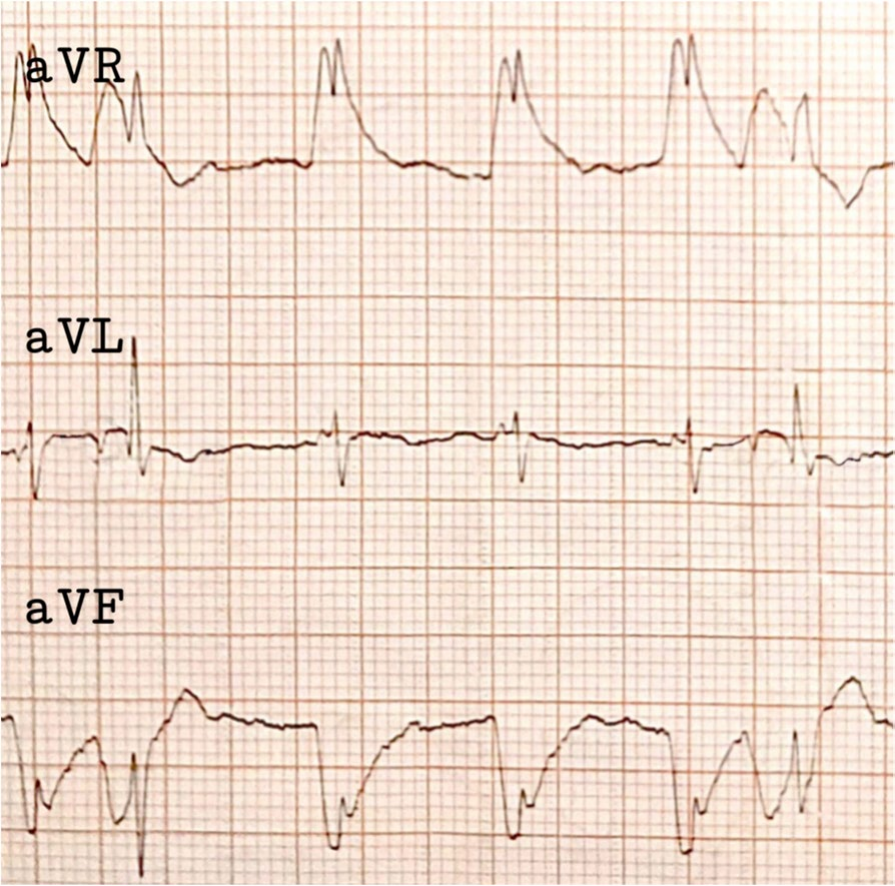
ECG identifying PACs on aVL electrode (middle) along with wave distortions on aVR (top) and aVF (bottom) electrodes

Electrocardiography (ECG) revealed a junctional rhythm with PACs occurring at a rate of 1 contraction every 4 beats on the aVL electrode, in addition to wave distortions on both the aVR and aVF electrodes (Fig. 3). Complete blood count, liver, and renal function tests yielded normal values. Electrolyte tests indicated a magnesium level of 1.7 mg/dl, which was corrected with a magnesium syrup.

**Fig. 4:**
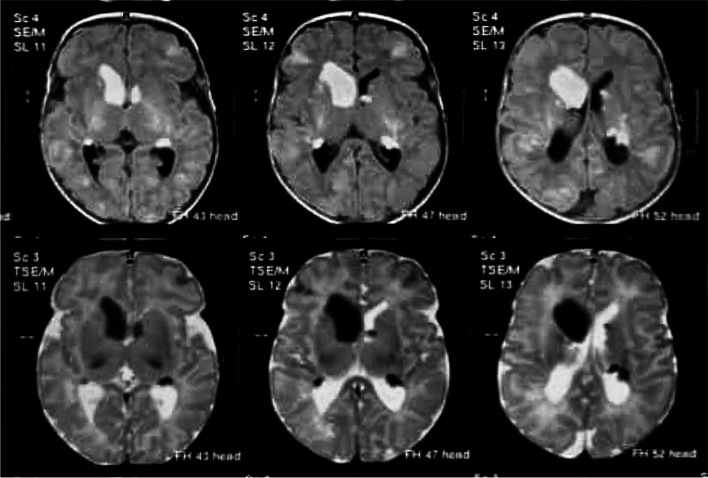
Axial T1-weighted and T2-weighted magnetic resonance images (MRI) showing high signal subepyndemal nodules at the frontal horn of the right lateral ventricle and in the occipital horns of both lateral ventricles

Further investigation with a brain magnetic resonance imaging (MRI) scan showed multiple subependymal nodules in the frontal horn of the right lateral ventricle and occipital horns of both lateral ventricles, which were characterized by high signal on both T1 and T2-weighted images (Fig. 4). The rest of the MRI results were not significant. Given these presenting findings and his family history, a preliminary diagnosis of TSC was suggested.

Anticonvulsants were prescribed. The patient remained asymptomatic until discharge. Daily cardiac investigations were conducted during his hospital stay. Since there were no major symptoms or hemodynamic instability, the patient was monitored through regular follow-ups. The patient has remained asymptomatic to date. He had a normal ECG at 9 months with no arrhythmias or PACs. Imaging prior to 9 months demonstrated signs of regression, but further imaging is required. The patient is scheduled for imaging in 2 months.

## Discussion and conclusions

Tuberous sclerosis complex is an autosomal dominant genetic disorder characterized by the formation of hamartomas in various organs such as the skin, brain, heart, lungs, kidneys, and liver [1]. It occurs sporadically in around two-thirds of patients [3]. De novo mutations result in the inactivation of the TSC1 gene located on chromosome 9q34.3 and the TSC2 gene located on chromosome 16p13.3, which are responsible for tuberin and hamartin production [3].

With a rare incidence of 0.2% [8], CRs are the most common type of benign cardiac tumors. CRs can occur spontaneously, in association with other congenital heart diseases, or in association with TSC [8], with approximately 60–80% of cases being linked to TS [2, 6, 9]. Józwiak et al. [4] examined 154 patients with TS and discovered that 74 (48%) of them had multiple CRs. These tumors were located in the right ventricle (35%), interventricular septum (33%), left ventricle (22%), left atrium (5%), and RA (5%). In our case, the RA, a rare location, contained a 25 × 20 mm rhabdomyoma (Fig. 2) that was later associated with TSC.

The clinical features of TSC consist of seizures, hydrocephalus, mental retardation, rhabdomyomas, renal angiomyolipomas, developmental delay, spine malformations, and skin angiofibromas [10]. CRs are usually multiple in more than 90% of cases [8], especially in the presence of tuberous sclerosis [11] and are typically asymptomatic. Nevertheless, arrhythmias, fatal outflow obstruction, and rarely myocardial infarction can occur in the womb [1]. ECGs can present a spectrum of different conduction defects, including tachycardia, bradycardia, ST segment changes, Wolff–Parkinson-White pre-excitation, and aberrant atrial or intraventricular conduction [12]. The prognosis depends on the size, location, and associated complications of the tumor [5]. Fetal cardiac tumors larger than 20 mm in diameter carry a higher risk of prenatal death [9]. In our scenario, the patient exhibited symptoms of irregular arrhythmias characterized by PACs in the presence of a solitary atrial mass; however, there were no signs of hemodynamic instability.

The diagnosis of rhabdomyoma can be determined through prenatal ultrasound or MRI during the third trimester of pregnancy or in the early neonatal period [1, 13]. Ultrasound findings typically show a round, uniform, hyperechogenic mass, primarily located in the ventricles [3]. Our echocardiography findings matched typical rhabdomyoma features. When considering the differential diagnosis, other potential conditions to consider are pericardial teratoma, fibroma, hemangioma, myxoma, and echogenic cardiac focus. The absence of pericardial effusion and prominent internal vascularity excludes teratoma and hemangioma. Differential diagnosis between the other tumors using ultrasonography alone is difficult [14]. However, given the patient’s family history and extracardiac findings that matched TSC, CR was the ideal clinical diagnosis.

The diagnosis of TSC relies on clinical criteria that are categorized as major and minor (Table 1) [15].

**Table 1:** Clinical criteria for diagnosing of tuberous sclerosis (2012)

Major features	Minor features
1. Hypomelanotic macules (≥3, at least 5-mm diameter)	1. “Confetti” skin lesions
2. Angiofibromas (≥3) or fibrous cephalic plaque	2. Dental enamel pits (> 3)
3. Ungual fibromas (≥2)	3. Intraoral fibromas (≥2)
4. Shagreen patch	4. Retinal achromic patch
5. Multiple retinal hamartomas	5. **Multiple renal cysts**
6. Cortical dysplasia	6. Nonrenal hamartomas
7. **Subependymal nodules**	
8. Subependymal giant cell astrocytoma	
9. **Cardiac rhabdomyoma**	
10. Lymphangioleiomyomatosis	
11. Angiomyolipomas (≥2)	

The diagnostic criteria of tuberous sclerosis according to the 2012 International Tuberous Sclerosis Complex Consensus Conference [15]. Bolded parts are observed in our case

To confirm the diagnosis, at least two major criteria or one major and two minor criteria must be present. A probable diagnosis can be made with one major criterion or two or more minor criteria [15]. Our case fulfilled two major criteria: cardiac rhabdomyoma and subependymal brain lesions, and one minor criterion, renal cysts. However, genetic analysis was not conducted in our case. Our diagnosis was obtained after birth. It is crucial to prenatally diagnose rhabdomyomas in order to investigate any association with TSC and plan appropriate treatment early on.

Regarding the treatment and management of TSC, significant progress has been made with mechanistic target of rapamycin complex 1 (mTORC1) inhibitors leading the way [16]. If the tumor obstructs normal cardiac outflow or leads to intractable arrhythmias, surgical intervention becomes a necessity [11, 17]. Typical rhabdomyoma cells lose their ability to divide and gradually regress during early childhood [11]. Postnatal echocardiograms should be done for at least a year to monitor the regression phase of rhabdomyomas and ensure there are no additional health risks for the infant [9]. In our patient, the tumor did not restrict blood flow, and further examination after 9 months revealed a hemodynamically stable patient with a normal ECG, and echocardiography demonstrated tumor regression.

### Supplementary Information

**Additional file 1:.**

**Additional file 2:.**

## Data Availability

Not applicable.

## Abbreviations

TSC: Tuberous sclerosis complex
CR: Cardiac rhabdomyoma
PAC: Premature atrial contraction
RA: Right atrium
