# Supplementary material for: Solitary atrial Rhabdomyoma in an infant with tuberous sclerosis: a case report and review of the literature
Source: BMC Cardiovasc Disord. 2023 Dec 7;23:597. doi: 10.1186/s12872-023-03639-4 (PMC10701948; doi:10.1186/s12872-023-03639-4)
Supplement: Supplementary file 1 — Additional file 1:. [file 12872_2023_3639_MOESM1_ESM.docx]

| **First Author** | **Year** | **Study design** | **Patient Age at diagnosis** | **F/M** | **Tumor/s Location** | **Cardiac Symptoms** | **Neurological Symptoms** | **TSC** | **Surgical treatment** |
| --- | --- | --- | --- | --- | --- | --- | --- | --- | --- |
| Sahin I(1) | 2022 | Case | 16 Years old | Male | LV | Yes | No | No | Death |
| Ban JE (2) | 2022 | Case | Neonate | Female | - | Yes | No | Yes | Death |
| Gosh S(3) | 2021 | Case | 34 Years old | Female | LV | No | No | Yes | Monitoring |
| Kondle VK(4) | 2021 | Case | 8 Years old | Female | - | No | Yes | Yes | Monitoring |
| Philip S(5) | 2021 | Case | Prenatal | - | LV | Yes | No | - | Pregnancy termination |
| Traisrisilp K (6) | 2021 | Case | Prenatal | - | - | No | Yes | No | - |
| Bassirou BM(7) | 2020 | Case | 3 months old | - | LV, RV | No | No | - | Monitoring |
| Prasad K(8) | 2020 | Case | Prenatal | - | LV, RV | Yes | No | Yes | Monitoring + Everolimus |
| Chen L(9) | 2020 | Case | Prenatal | - | RV | - | - | Yes | Pregnancy termination |
| Poonia A(10) | 2020 | Case | Neonate | - | LV, RV | Yes | No | - | Surgical |
| Frudit P(11) | 2019 | Case | 7-day old | Female | LV | No | No | Yes | Death |
| Garg A(12) | 2019 | Case | Prenatal | - | RV | Yes | No | Yes | Drug therapy (Everolimus) |
| Wu C(13) | 2019 | Case | Prenatal | Female | LV, LA | Yes | No | No | Surgical |
| Sarrf B(14) | 2019 | Case | Neonate | Male | LV, RV | No | No | Yes | Monitoring |
| Song ES(15) | 2018 | Case | Neonate | Male | LV, RV | Yes | No | Yes | Monitoring |
| Ergül Y(16) | 2018 | Case | 8-day old neonate | - | - | Yes | No | - | Amiodarone and propafenone |
| Ruan W(17) | 2017 | Case | 5 months | Female | - | Yes | No | - | Monitoring |
| Kayali S(18) | 2017 | Case | Neonate | Female | LV | Yes | No | No | Everolimus, Surgical |
| Chinawa JM(19) | 2017 | Case | 2.5 months | Male | RV | Yes | No | No | Monitoring |
|  |  |  | 8 months | Male | RV | No | No | Yes | Monitoring |
| Hutchinson JC(20) | 2016 | Case | Neonate | - | LV, RV | Yes | No | Yes | Death |
| Cotaina GL(21) | 2016 | Case | Neonate | Male | RV | / | / | Yes | Surgical |
| Meshram R(22) | 2015 | Case | Neonate | Male | LV | Yes | No | - | - |
| [Thatte](https://www.cambridge.org/core/journals/cardiology-in-the-young/article/abs/newonset-cardiac-rhabdomyoma-beyond-infancy-in-a-patient-with-tuberous-sclerosis-complex/8079C5A047FD30850BC4049FA99409CC) NM(23) | 2015 | Case | Neonate, recurrence at 2 years | Male | RV | Yes | No | Yes | Surgical |
| Jurko T(24) | 2015 | Case | Prenatal | Male | LV | No | Yes | Yes | Monitoring |
|  |  |  | Prenatal | Male | LV | Yes | No | Yes | Monitoring |
|  |  |  | Prenatal | Female | LV | No | Yes | Yes | Monitoring |
| Bondavalli D(25) | 2015 | Case | 5 months | Male | LV | Yes | No | No | Not mentioned |
| Sadoh WE(26) | 2014 | Case | Neonate | Male | LV | Yes | No | No | Monitoring |
| Shen Q(27) | 2014 | Case-series | 4 months | Male | LV, RV | No | Yes | Yes | Monitoring |
|  |  |  | 3 months | Female | LV, RV | Yes | Yes | Yes | Monitoring |
|  |  |  | 5 months | Male | LV, RV, LA | Yes | Yes | Yes | Surgical |
|  |  |  | 5 years | Male | LV | No | Yes | Yes | Monitoring |
|  |  |  | 6 months | Male | LV | Yes | Yes | Yes | Monitoring |
|  |  |  | 10 months | Male | LV, RV | No | Yes | Yes | Monitoring |
|  |  |  | 2 years | Male | RV | No | Yes | Yes | Monitoring |
| Azhari N(28) | 2014 | Case | 3 weeks | male | RV | Yes | No | Yes | Monitoring |
| El-Segaier M(29) | 2014 | Case | 1 month | - | LV | Yes | No | - | Surgical |
| Castilla Cabanes E(30) | 2014 | Case | 12-day old | Female | LV, RV | Yes | No | Yes | Monitoring |
| Kocabaş A(31) | 2013 | Retrospective | Neonate | Female | RV, RA | Yes | 7 out of 11 | Yes | Surgical |
|  |  |  | Neonate | Male | LV | Yes |  |  | Medical |
|  |  |  | Prenatal | Female | RV, LV | Yes |  |  | Monitoring |
|  |  |  | Prenatal | Male | LV | Yes |  |  | Monitoring |
|  |  |  | 2 months | Male | RV | Yes |  |  | Surgical |
|  |  |  | 7 months | Male | RV, LV | Yes |  |  | Monitoring |
|  |  |  | 3.5 months | Male | RV | No |  |  | Monitoring |
|  |  |  | 6.5 months | Female | LV | No |  |  | Monitoring |
|  |  |  | 6.5 months | Male | RV | No |  |  | Monitoring |
|  |  |  | 10.5 months | Male | RV, LV | No |  |  | Monitoring |
|  |  |  | 12 months | Female | LV | No |  |  | Monitoring |
| Kutluk T(32) | 2013 | Case-Series | Neonate | Female | LV, RV | Yes | - | 1 out of 6 | Monitoring |
|  |  |  | Neonate | Male | LV | Yes |  |  | Monitoring |
|  |  |  | Neonate | Male | LV | No |  |  | Monitoring |
|  |  |  | Neonate | Female | LV | Yes |  |  | Surgical |
|  |  |  | Neonate | Female | RV | Yes |  |  | Monitoring |
|  |  |  | Neonate | Female | RV | Yes |  |  | Surgical |
| Chaurasia AK(33) | 2013 | Case | 5 months old | Female | RV | No | No | Yes | Monitoring |
| Walsh K(34) | 2013 | Observational | 9 antenatal cases | - | "Most" in the LV | 7 out of 9 | "Most" Yes | Yes | Death |
|  |  |  |  |  |  |  |  |  | Monitoring |
|  |  |  | Post natal (12) |  |  | 4 out of 12 |  |  |  |
|  |  |  | - |  |  | - |  |  |  |
| Karatas A(35) | 2013 | Case | Prenatal | Female | LV,RV | No | no | No | monitoring |
| Benyounes N(36) | 2012 | Case | 10 years | Female | RV | yes | no | Yes | Monitoring |
| Pruksanusak N (37) | 2012 | Case | Prenatal | Male | LV, RV | No | Yes | - | Death |
|  |  |  | Prenatal | - | LV, RV | Yes | - | Yes | Death |
| Kaushik SK(38) | 2012 | Case | Neonate | Male | LV,RV | No | Yes | No | Surgical |
| Knight CJ(39) | 2012 | Case | Prenatal | - | LV | Yes | Yes | Yes | Monitoring |
| Courand PY(40) | 2012 | Case | 28 years old | Female | LV | No | No | Yes | Death |
| Morales-Quispe J(41) | 2011 | Case | Prenatal | male | LV, RV | No | No | - | Death |
| Madueme P(42) | 2011 | Case | 3 months old | male | LV, RV | No | Yes | Yes | Monitoring |

**Legend:**

**Retrieved cases of cardiac rhabdomyomas**

All case reports and case-series including patients with cardiac rhabdomyomas (CR) were retrieved from PUBMED from March 2011 to January 2023. A total of 88 cases were identified. Excluding not mentioned (-) results, 82% of CRs were associated with tuberous sclerosis. 73.8% of CRs occurred in the left ventricle (LV), 59% in the right ventricle, and only 2% (1 out of 61) in the RA. Around 58% (50 out of 86) had cardiac symptoms (arrythmias, bradycardia, tachycardia, cyanosis, hemodynamic instability, and murmurs), whereas 38% (22 out of 57) had neurologic symptoms (seizures, intellectual disability). Only 12% of cases required surgical intervention, whereas 79% underwent monitoring and sometimes needed medical therapy (everolimus, antiarrhythmics, and antipsychotics). 9% of cases (8 out of 85) died due to complications.

- = Not mentioned.

**Referenced cases and case-series:**

1. Sahin I, Yucel SM, Yüksel Ş. A Rare Presentation of Cardiac Rhabdomyoma in Children: Sudden Cardiac Arrest. In 2022 [cited 2023 Sep 23]. Available from: https://www.semanticscholar.org/paper/A-Rare-Presentation-of-Cardiac-Rhabdomyoma-in-Sahin-Yucel/2e636a38c1a88c33885787f7b224c8caa40bef12

2. Ban JE. Response to Letter to the Editor: Giant Cardiac Rhabdomyoma with Mixed Atrial Tachycardia and Nonsustained Ventricular Tachycardia in a Newborn with Tuberous Sclerosis. Ewha Med J [Internet]. 2023 [cited 2023 Sep 23];46(2). Available from: http://www.e-emj.org/archive/view_article?pid=emj-46-2-5

3. Ghosh S, Milunski MR. Cardiac Rhabdomyoma in Adult. Cureus. 13(4):e14565.

4. Kondle VK, Padugundla G, Akshitha P, Rajaboina V. Cardiac Rhabdomyoma with Tuberous Sclerosis - A Case of Unresponsive Seizures. Telangana J IMA. 2021 Oct 29;1(2):46–9.

5. Philip S, Thampy L. A Solitary Fetal Cardiac Rhabdomyoma: A Hemodynamically Unstable Left Ventricular Tumor with Autopsy and Histopathology Findings. J Fetal Med. 2021 Jun 1;8(2):163–8.

6. Traisrisilp K, Sirikunalai P, Sirilert S, Chareonsirisuthigul T, Tongsong T. Cardiac rhabdomyoma as a possible new prenatal sonographic feature of Prader–Willi syndrome. J Obstet Gynaecol Res. 2022;48(1):239–43.

7. Bassirou BM, Mohamed L, Fatou A, Alassane NA, Abdoulaye C, Yaya BE, et al. Infant Cardiac Rhabdomyoma: About a Case. (657).

8. Prasad K, Barwad P, R PC, Santosh K, Vijay J, Naganur S. Accelerated regression of cardiac rhabdomyoma by mTOR inhibitors in a neonate with heart failure: A case report. IHJ Cardiovasc Case Rep CVCR. 2020 Sep 1;4(3):142–5.

9. Chen L, Jiang Y, Wang J. Fetal cardiac rhabdomyoma due to paternal mosaicism of TSC2. Medicine (Baltimore). 2020 Aug 28;99(35):e21949.

10. Poonia A, Giridhara P, Gopalakrishnan A. Cardiac Rhabdomyoma Causing Progressive Dynamic Severe Right Ventricular Outflow Tract Obstruction in an Infant. Indian Pediatr. 2020 Feb 15;57(2):179–80.

11. Frudit P, Vitturi BK, Navarro FC, Rondelli I, Pozzan G. Multiple cardiac rhabdomyomas in tuberous sclerosis complex: case report and review of the literature. Autopsy Case Rep. 2019 Sep 30;9(4):e2019125.

12. Garg A, Gorla SR, Kardon RE, Swaminathan S. Rapid Involution of Large Cardiac Rhabdomyomas With Everolimus Therapy. World J Pediatr Congenit Heart Surg. 2021 May 1;12(3):426–9.

13. Wu C, Wang Y, Su W, Xia J, Dong N, Wang G. Cardiac rhabdomyomas with atrial septal defect and tricuspid insufficiency: A case report. J Card Surg. 2019 Oct;34(10):1123–6.

14. Sarff B, Floyd R, Bildner A, Stormo J, Fisher K. Fetal Echocardiographic Detection of Cardiac Tumors: A Case Report of Multiple Fetal Cardiac Rhabdomyomas. J Diagn Med Sonogr. 2019 Sep 1;35(5):426–30.

15. Song ES, Jeong K, Kim G, Hwang IJ, Lee MJ, Cho HJ, et al. Spontaneous Regression of Cardiac Rhabdomyoma Presenting as Severe Left Ventricular Inlet Obstruction in a Neonate with Tuberous Sclerosis. Case Rep Cardiol. 2018 Jan 28;2018:e8395260.

16. Ergül Y, Öztürk E, Güzeltaş A. Atrial flutter and nonconducted bigeminy premature atrial contraction in a neonate with cardiac rhabdomyoma. Turk Kardiyol Dernegi Arsivi Turk Kardiyol Derneginin Yayin Organidir. 2018 Jun;46(4):313–7.

17. Ruan W, Han J, Wang Y, Lin K. Cardiac rhabdomyoma in an infant patient presenting with right ventricular outflow tract obstruction. J Card Surg. 2017 Jan;32(1):62–3.

18. Kayali S, Dogan V, Arda N, Koç M, Ertugrul I, Özgür S, et al. Symptomatic Fetal-Type Cardiac Rhabdomyoma. J Coll Physicians Surg--Pak JCPSP. 2017 Mar;27(3):S53–5.

19. Chinawa JM, Garekar S, Trivedi B. Large cardiac rhabdomyoma in two infants: Good response to medical management – A brief report. Niger J Cardiol. 2017 Dec;14(2):106.

20. Hutchinson JC, Ashworth MT, Sebire NJ, Arthurs OJ. Multiple Cardiac Rhabdomyomas Visualised Using Micro-CT in a Case of Tuberous Sclerosis. Fetal Diagn Ther. 2017;41(2):157–60.

21. Cotaina GL, Lázaro GE, Jiménez MI, Savirón CR, Lerma PD. [Diagnosis of cardiac rhabdomyoma in the first trimester of pregnancy]. Ginecol Obstet Mex. 2016 Mar;84(3):180–5.

22. Meshram R, Gondase S, Denge A, Kamble N. Huge cardiac rhabdomyoma in neonate: an unusual presentation. Int J Contemp Pediatr. 2020 Oct 21;7:2249.

23. Thatte NM, Guleserian KJ, Reddy SRV. New-onset cardiac rhabdomyoma beyond infancy in a patient with tuberous sclerosis complex. Cardiol Young. 2016 Feb;26(2):396–9.

24. Jurko T, Jurko A, Krsiakova J, Jurko A, Minarik M, Matasova K, et al. Prenatal diagnosis of cardiac rhabdomyoma associated with tuberous sclerosis: report of 3 cases. Neuro Endocrinol Lett. 2015 Dec;36(6):521–3.

25. Bondavalli D, White SM, Steer A, Pflaumer A, Winship I. Is cardiac rhabdomyoma a feature of Birt Hogg Dubé syndrome? Am J Med Genet A. 2015 Apr;167(4):802–4.

26. Sadoh WE, Obaseki DE, Amuabunos EA, Eregie CO, Isah IA, Idemudia E, et al. Cardiac Rhabdomyoma in a Neonate With Supraventricular Tachycardia. World J Pediatr Congenit Heart Surg. 2014 Jan;5(1):110–3.

27. Shen Q, Shen J, Qiao Z, Yao Q, Huang G, Hu X. Cardiac rhabdomyomas associated with tuberous sclerosis complex in children: From presentation to outcome. Herz. 2015 Jun;40(4):675–8.

28. Azhari N, Hakim M, Barefah G. Cardiac rhabdomyoma presenting as infective endocarditis: a case report. Cardiol Young. 2015 Mar;25(3):557–9.

29. El-Segaier M, Galal MO. Cardiac rhabdomyoma mimicking haemodynamics of hypoplastic left heart syndrome. Acta Cardiol. 2014 Jun;69(3):308–10.

30. Castilla Cabanes E, Lacambra Blasco I. Multiple Cardiac Rhabdomyomas, Wolff-Parkinson-White Syndrome, and Tuberous Sclerosis: An Infrequent Combination. Case Rep Pediatr. 2014;2014:1–4.

31. Kocabaş A, Ekici F, Cetin Iİ, Emir S, Demir HA, Arı ME, et al. Cardiac rhabdomyomas associated with tuberous sclerosis complex in 11 children: presentation to outcome. Pediatr Hematol Oncol. 2013 Mar;30(2):71–9.

32. Kutluk T, Demir HA, Büyükpamukçu M, Ozkutlu S, Akyüz C, Varan A, et al. Cardiac rhabdomyomas in childhood: six cases from a single institution. Turk J Pediatr. 2013;55(1):69–73.

33. Chaurasia AK, Harikrishnan S, Bijulal S, Choudhary D, Tharakan J. Cardiac Rhabdomyoma In Familial Tuberous Sclerosis. J Cardiovasc Thorac Res. 2013;5(2):71–2.

34. Walsh M, Naude JTW, Uzun O. PF.56 Cardiac Rhabdomyomas in Fetal Life and Beyond: A Single Centre 15-Year Experience. Arch Dis Child - Fetal Neonatal Ed. 2013 Apr 1;98(Suppl 1):A19–20.

35. Karatas A, Karatas Z, Ozlu T. Fetal cardiac rhabdomyoma without Tuberous Sclerosis: A case report. Int J Med Sci Public Health. 2013 Jan 1;2:1095.

36. Benyounes N, Fohlen M, Devys JM, Delalande O, Moures JM, Cohen A. Cardiac rhabdomyomas in tuberous sclerosis patients: a case report and review of the literature. Arch Cardiovasc Dis. 2012;105(8–9):442–5.

37. Pruksanusak N, Suntharasaj T, Suwanrath C, Phukaoloun M, Kanjanapradit K. Fetal Cardiac Rhabdomyoma With Hydrops Fetalis: Report of 2 Cases and Literature Review. J Ultrasound Med. 2012 Nov;31(11):1821–4.

38. Kaushik SK, Bhargava K, kaushik A. Cardiac rhabdomyoma with LVOT obstruction and anorectal malformation in a neonate: A rare association. Indian Heart J. 2012 Sep 1;64(5):508–10.

39. Knight CJ, Pembridge JM. Cardiac rhabdomyoma presenting with fetal bradycardia: Is immediate delivery always the answer? J Obstet Gynaecol. 2012 May;32(4):399–399.

40. Courand PY, Barthelet M, Cordier JF, Cottin V. Persistent cardiac rhabdomyoma in an adult with tuberous sclerosis. Eur Heart J - Cardiovasc Imaging. 2012 Jul 1;13(7):567.

41. Morales-Quispe J, Zavaleta N, Caballero-Caballero R, Brunner-Cruz G, Alcántara S. Multiple cardiac rhabdomyoma associated to intrauterine death. Arch Cardiol México. 2011 Jul 1;81:217–20.

42. Madueme P, Hinton R. Tuberous Sclerosis and Cardiac Rhabdomyomas: A Case Report and Review of the Literature: Tuberous Sclerosis and Cardiac Rhabdomyomas. Congenit Heart Dis. 2011 Mar;6(2):183–7.
